# Aquaponic growth of basil (*Ocimum basilicum*) with African catfish (*Clarias gariepinus*) in standard substrate combined with a Humicacid Fiber-Substrate (HFS)

**DOI:** 10.1038/s41598-024-68361-3

**Published:** 2024-07-31

**Authors:** Ulrich Knaus, Dirk Hyo-Dschung Hübner, Christian Küchenmeister, Samuel Appelbaum, Walter Iten, Harry W. Palm

**Affiliations:** 1 Faculty of Agricultural and Environmental Sciences, Professor of Aquaculture and Sea-Ranching, University of Rostock, Justus-von-Liebig-Weg 6, 18059 Rostock, Germany; 2 French Associates Institute for Agriculture and Biotechnology of Drylands, Jacob Blaustein Institutes for Desert Research, Ben-Gurion University of the Negev, Sede Boqer Campus, 8499000 Midreshet Ben-Gurion, Israel; 3 Institut für angewandte Naturwirtschaft/Natural Science IfaN GmbH, Sennweidstrasse 44, 6312 Steinhausen, Switzerland

**Keywords:** Aquaponics farming, Basil, African catfish, Biologically-pure-three-phase-natural-fertiliser, Humicacid Fiber-Substrate (HSF), Plant domestication, Biological techniques

## Abstract

A major challenge in agriculture, horticulture and aquaponics practices is the reduction of mineral fertilisers and peat to reduce CO_2_ emissions and increase sustainability. This study used a three-phase-natural fertiliser, the Humicacid Fiber-Substrate (HFS), made from natural regenerative organic and mineral-fractions (Humus-Mineral-Complex), to reduce the peat content in plant pots for aquaponics farming. Basil (*Ocimum basilicum*) growth was compared with *i*) 100% standard media substrate ("Einheitserde", white peat 80%, clay 20%), and *ii*) 85% "Einheitserde" and 15% of HFS under irrigation with aquaculture process waters from an extensive and intensive production of African catfish (*Clarias gariepinus*) under coupled aquaponic conditions. The substitution with 15% HFS and use of intensive fish water resulted in comparable plant growth to a fertiliser solution as control, and in higher leaf width and leaf green weight and lower root dry weight compared with the standard media substrate "Einheitserde". Basil leaf chlorophyll content from the aquaponics was higher compared with local market plants. This suggests the possible substitution of the peat substrate "Einheitserde" with at least 15% HFS to reduce the natural peat fraction. Further studies on crop-specific substrates are needed to reduce peat in aquaponics farming plant cultivation.

## Introduction

The reduction of commercial mineral fertilisers is one of the most important advantages of aquaponics and depends on the fish feed-based nutrient input^1^, which is mainly influenced by the fish species and variations in fish stocking density^2^. Also, hydroponic components, such as "grow pipes" or "aeroponic systems", influence the plant growth^3–6^. Aquaponics (*s.l.*) farming as pot cultivation is a relatively new technique for e.g. spearmint (*Mentha spicata*)^7^. The potting substrate provides supplementary nutrients when applying aquaponics horticultural techniques^2^. Thus, the supplementary "nutrient substrate" should best originate from the circular economy in the form of agricultural by-products to ensure highest ecological benefits^8^. The reuse of nutrients from agricultural biomass or "lost material"^9^ can contribute to a more sustainable production ("nutrient cascade"). Phosphorus and nitrogen reuse from agricultural by-products for crop production directly reduces the demand for fertiliser from mineral and fossil raw materials and returns the nutrients back into the agricultural economy^10^.

Substrates for hydroponic crop production mainly contain peat moss (*Sphagnum magellanicum*)^11^. The standard garden pot substrate, according to Fruhstorfer (German: "Fruhstorfer Erde" or "Einheitserde"), is a mixture of decomposed raised bog peat (white peat, with more than 94% organic matter) and clay^12,13^. As a substrate starting material, bog peat has positive properties, such as a low pH value, a low content of plant-available nutrients, low microbial activity, and a good water and air supply; it can be fertilised individually according to the plant needs^12^. However, in order to replace peat, alternative growth substrates are needed that allow similar growth performance. Soil conditioners consist of organic and inorganic residual materials such as greens, wood, and compost (municipal, forestry, agricultural) as well as by-products from the processing industry e.g., burned clay, slaked lime or wine waste, and have been used as adsorbents for heavy metals, for increasing the nutrient contents, and for improving soil properties^14–17^. A soil regenerator (soil conditioner) was patented in the early 1990’s under the title "Biologically pure, three-phase natural fertilizer and process for producing the same" (WO1993010061A1, Switzerland, 1992)^18^, consisted of the three fractions a) crushed igneous rock, b) brown coal and c) mussel lime and optional different seeds. In a more recent design (green stuff), local plant material was combined with the mineral components. The resulted was a homogeneous industry made rock/plant/humus fiber substrate (humus equivalent) of uniform quality with all necessary micro- and macroelements, comparable to natural black peat, and was already successfully applied in the desert of Jordan^19^.

African catfish (*C. gariepinus*) is a popular food fish in Mecklenburg-Western Pomerania (Northern Germany), and the production of six aquaculture farms reached a yield of 883 t in 2020, representing 76% of the total aquaculture production in this federal state^20^. This species was newly introduced around 2010 to aquaponics, resulting in good feed conversion ratios (FCRs), ranging from 0.94 to 0.96 when cultivated with spearmint (*Mentha spicata*)^7^, and 1.23–1.39 when cultivated with water spinach (*Ipomoea aquatica*)^21^. Good FCR values of *C. gariepinus*, ranging from 0.97 to 1.12 were also achieved when iron (FeSO_4_) was added to the process water under aquaponic conditions^22^. These studies demonstrated *C. gariepinus* as a useful fish species for coupled aquaponics. Basil (*Ocimum basilicum*, Lamiaceae) was identified as a high-value, fast-growing herb, and was successfully applied in aquaponics with Nile tilapia (*Oreochromis niloticus*)^23^, African catfish (*Clarias gariepinus*)^4,5^, carp (*Cyprinus carpio*)^24^, and crayfish (*Procambarus* spp.)^25^.

In the present study, the cultivation of basil (*O. basilicum*) was evaluated in combination with African catfish (*C. gariepinus*) as aquaponics farming *sensu lato* (*s.l.*) according to Palm et al. in horticulture^2^. The growth of basil was compared in two trials with (*i*) a proportion of 100% standard growth media substrate "Einheitserde" (E; Abbreviations) as a potting substrate, and (*ii*) using a soil conditioner, the Humicacid Fiber-Substrate (HFS) with a content of 15% in the pots and 85% “Einheitserde”. The pot cultures were irrigated with aquaculture wastewater from extensive (EAU) and intensive (IAU) African catfish production (Fig. 4) and compared with a commercially available hydroponic fertiliser at a standardised EC of approx. 2000 µS/cm as a control (C) with 100% standard growth media substrate "Einheitserde" and an addition of dried commercial start fertiliser. The influence of HFS on the growth performance of basil and the change in the nutrient composition of the substrates is discussed.

## Results

### Trial I: Basil production with standard growth media substrate (Einheitserde)

Growth of *O. basilicum* in 100% standard growth media substrate (“Einheitserde”) showed the highest values in the control and comparable levels in the EAU and IAU (Table 1). Plant height was significantly higher in the control (61.9 ± 8.5 cm), followed by the IAU (48.5 ± 5.0 cm) and EAU (45.6 ± 4.3 cm). Root length was highest in the control (18.9 ± 2.6 cm) and not significantly different to the intensive aquaculture process water (17.9 ± 2.0 cm), which was comparable to the extensive process water (16.9 ± 2.6 cm). In contrast, root dry weight was significantly different between the groups with the highest value in the control (1.1 ± 0.2 g), followed by the IAU (0.5 ± 0.1 g) and EAU (0.4 ± 0.1 g). Leaf number was highest in the control (71.5 ± 12.0) and similar in the IAU (39.7 ± 5.8) and EAU (36.3 ± 5.8). Leaf length was significantly different between the groups with the order as follows: control (12.0 ± 0.7 cm), IAU (10.7 ± 0.5 cm), and EAU (10.1 ± 0.6 cm). Leaf green weight showed the best value in the control (1.2 ± 0.2 g), followed by EAU (1.0 ± 0.1 g) and IAU (0.9 ± 0.1 g).

**Table 1:** The *O. basilicum* growth parameters (mean ± SD; n = 33) and SPAD (n = 18) cultured in pots of control with commercial substrate (S1: 100% “Einheitserde”: E, + dried starter fertiliser, Trial I) and irrigated with hydroponic fertiliser solution (Control + S1), and basil cultured in pots with 100% standard growth media substrate (“Einheitserde”, E) with irrigation of extensive (EAU) and intensive (IAU) aquaculture process water after 45 days; different letters showing different groups (p < 0.05; *p*-I: significance value (*p*) between Control & EAU; *p*-II: between Control & IAU; *p*-III: between EAU and IAU).

Parameters	Control + S1	Extensive (E + EAU)	Intensive (E + IAU)	*p*-I	*p*-II	*p*-III
Plant height (cm)	61.9 ± 8.5^a^	45.6 ± 4.3^b^	48.5 ± 5.0^b^	0.001	0.001	0.078
Green weight (g)	72.3 ± 14.2^a^	24.9 ± 4.7^b^	29.1 ± 5.7^b^	0.001	0.001	0.149
Dry weight (g)	6.8 ± 1.3^a^	2.4 ± 0.4^c^	2.7 ± 0.5^b^	0.001	0.001	0.018
Shoot length (cm)	43.0 ± 7.7^a^	28.7 ± 3.2^b^	30.7 ± 4.5^b^	0.001	0.001	0.153
Shoot green weight (g)	58.5 ± 12.6^a^	19.6 ± 3.2^c^	22.2 ± 4.4^b^	0.001	0.001	0.021
Shoot dry weight (g)	5.7 ± 1.2^a^	2.0 ± 0.4^b^	2.2 ± 0.5^b^	0.001	0.001	0.137
Root length (cm)	18.9 ± 2.6^a^	16.9 ± 2.6^b^	17.9 ± 2.0^ab^	0.003	0.206	0.210
Root green weight (g)	13.8 ± 4.5^a^	5.3 ± 2.5^b^	6.9 ± 2.0^b^	0.001	0.001	0.093
Root dry weight (g)	1.1 ± 0.2^a^	0.4 ± 0.1^c^	0.5 ± 0.1^b^	0.001	0.001	0.004
Shoot/root ratio dry weight (g)	5.4 ± 1.4^a^	5.3 ± 1.3^a^	4.4 ± 0.9^b^	1.000	0.005	0.015
Shoot/root ratio length (cm)	2.3 ± 0.6^a^	1.7 ± 0.3^b^	1.7 ± 0.3^b^	0.001	0.001	1.000
Leaf number (no)	71.5 ± 12.0^a^	36.3 ± 5.8^b^	39.7 ± 5.8^b^	0.001	0.001	0.343
Leaf width (cm)	7.7 ± 0.6^a^	7.1 ± 0.5^b^	7.3 ± 0.3^b^	0.001	0.005	0.198
Leaf length (cm)	12.0 ± 0.7^a^	10.1 ± 0.6^c^	10.7 ± 0.5^b^	0.001	0.001	0.001
Leaf green weight (g)	1.2 ± 0.2^a^	1.0 ± 0.1^b^	0.9 ± 0.1^c^	0.001	0.001	0.005
SPAD (%)	39.5 ± 4.2^a^	33.6 ± 4.3^b^	33.1 ± 4.2^b^	0.001	0.001	0.923

### Trial II: Basil production with Humicacid Fiber-Substrate (HFS)

*O. basilicum* growth parameters were significantly higher in the control than in the aquaponics groups cultured with 15% HFS (Table 2). Plant height was highest in the control (61.2 ± 5.5 cm), followed by the IAU (48.2 ± 5.4 cm) and EAU (40.5 ± 3.8 cm). The same pattern was found in green weight (control: 56.2 ± 10.0 g; IAU: 27.9 ± 6.0 g; EAU: 19.5 ± 5.6 g), dry weight (control: 5.2 ± 0.9 g; IAU: 2.5 ± 0.6 g; EAU: 1.6 ± 0.4 g), and leaf number (control: 60.6 ± 6.8; IAU: 38.2 ± 8.3; EAU: 26.6 ± 5.6), respectively. Comparable values were found between the IAU and EAU in root green weight (EAU: 6.3 ± 2.5 g; IAU: 6.8 ± 2.2 g) and root dry weight (EAU: 0.4 ± 0.1 g; IAU: 0.4 ± 0.1 g). The SPAD level was significantly higher in the control (37.3 ± 3.0%) and similar in the EAU (33.6 ± 3.1%) and IAU (33.9 ± 3.1%). Comparable values between the control and the IAU were found in root length (control: 18.6 ± 3.1 cm; IAU: 17.4 ± 2.0 cm) and leaf width (control: 7.7 ± 0.5 cm; IAU: 7.5 ± 0.6 cm).

**Table 2:** The *O. basilicum* growth parameters (mean ± SD; n = 33) and SPAD (n = 18) cultured in pots of control with commercial substrate (S2: 100% “Einheitserde”: E, + dried starter fertiliser, Trial II) and irrigated with hydroponic fertiliser solution (Control + S2), and basil cultured in pots with 15% Humicacid Fiber-Substrate (HFS) and 85% standard growth media substrate (Einheitserde) with irrigation of extensive (EAU) and intensive (IAU) aquaculture process water after 45 days; different letters showing different groups (p < 0.05; *p*-I: significance value (*p*) between Control & EAU; *p*-II: between Control & IAU; *p*-III: between EAU and IAU).

Parameters	Control + S2	Extensive (HFS + EAU)	Intensive (HFS + IAU)	*p*-I	*p*-II	*p*-III
Plant height (cm)	61.2 ± 5.5^a^	40.5 ± 3.8^c^	48.2 ± 5.4^b^	0.001	0.001	0.001
Green weight (g)	56.2 ± 10.0^a^	19.5 ± 5.6^c^	27.9 ± 6.0^b^	0.001	0.001	0.001
Dry weight (g)	5.2 ± 0.9^a^	1.6 ± 0.4^c^	2.5 ± 0.6^b^	0.001	0.001	0.001
Shoot length (cm)	42.6 ± 4.3^a^	23.6 ± 4.0^c^	30.8 ± 4.6^b^	0.001	0.001	0.001
Shoot green weight (g)	45.8 ± 8.8^a^	13.3 ± 3.8^c^	21.1 ± 5.0^b^	0.001	0.001	0.001
Shoot dry weight (g)	4.4 ± 0.8^a^	1.3 ± 0.3^c^	2.0 ± 0.6^b^	0.001	0.001	0.001
Root length (cm)	18.6 ± 3.1^a^	16.9 ± 1.6^b^	17.4 ± 2.0^ab^	0.020	0.179	0.617
Root green weight (g)	10.4 ± 6.0^a^	6.3 ± 2.5^b^	6.8 ± 2.2^b^	0.001	0.003	1.000
Root dry weight (g)	0.8 ± 0.2^a^	0.4 ± 0.1^b^	0.4 ± 0.1^b^	0.001	0.001	0.224
Shoot/root ratio dry weight (g)	6.0 ± 1.7^a^	3.9 ± 2.3^c^	4.8 ± 1.4^b^	0.001	0.038	0.007
Shoot/root ratio length (cm)	2.4 ± 0.5^a^	1.4 ± 0.3^c^	1.8 ± 0.3^b^	0.001	0.001	0.003
Leaf number (no)	60.6 ± 6.8^a^	26.6 ± 5.6^c^	38.2 ± 8.3^b^	0.001	0.001	0.003
Leaf width (cm)	7.7 ± 0.5^a^	6.8 ± 0.4^b^	7.5 ± 0.6^a^	0.001	0.893	0.001
Leaf length (cm)	11.8 ± 0.7^a^	9.4 ± 0.7^c^	10.7 ± 0.7^b^	0.001	0.001	0.001
Leaf green weight (g)	1.1 ± 0.1^a^	0.7 ± 0.1^c^	0.9 ± 0.1^b^	0.001	0.001	0.001
SPAD (%)	37.3 ± 3.0^a^	33.6 ± 3.1^b^	33.9 ± 3.1^b^	0.001	0.004	0.940

### Comparison of basil growth with standard growth media substrate (Einheitserde, E, Trial I) and Humicacid Fiber-Substrate (HFS, Trial II)

**Figure 1:**
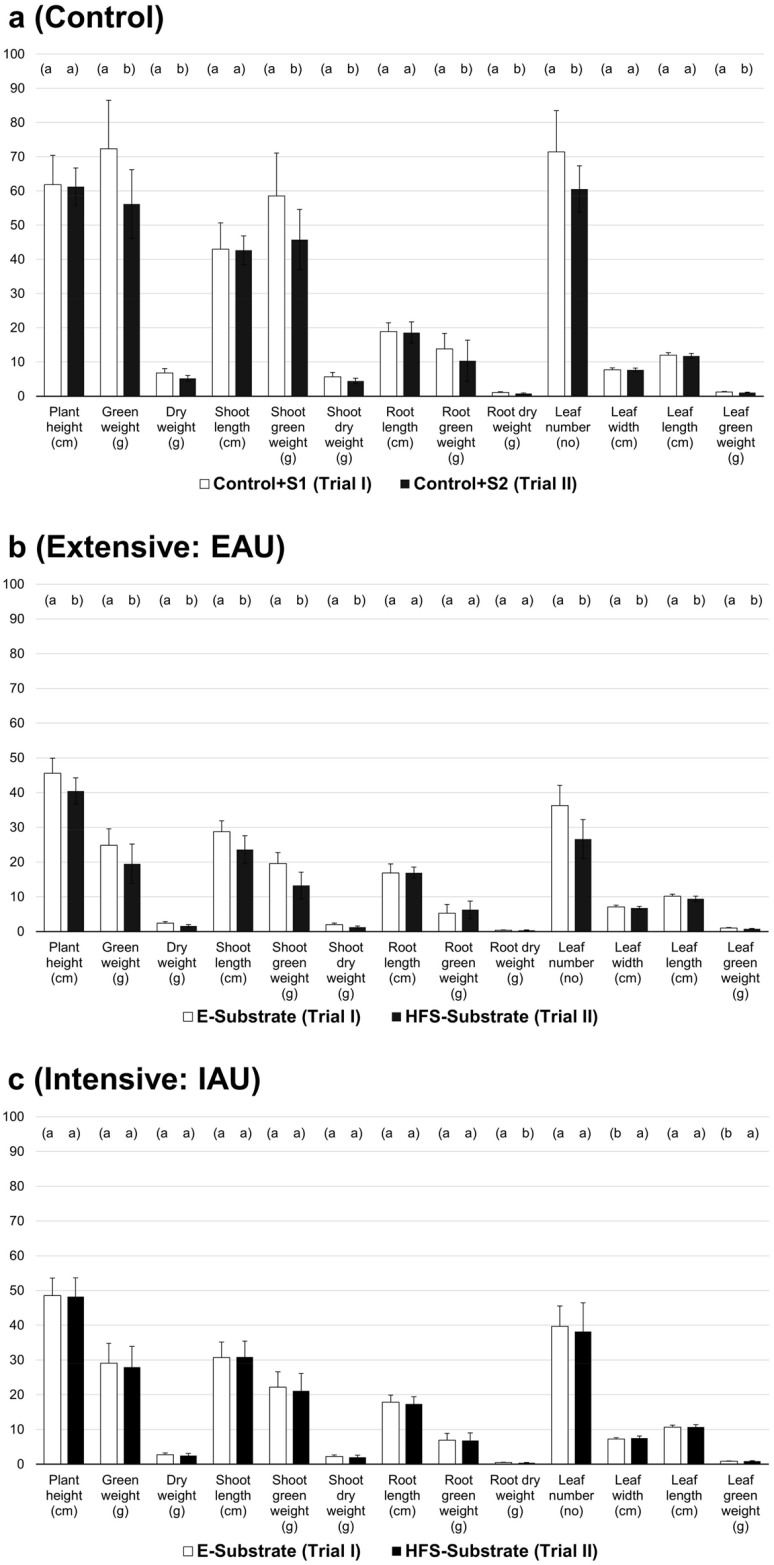
(**a**–**c**) Comparison of *O. basilicum* growth parameters between (**a**) control with commercial standard growing media substrate in each pot (S1 of Trial I and S2 of Trial II: 100% Einheitserde + dried commercial starter fertiliser) of plants irrigated with hydroponic fertiliser solution (Universol^®^ Orange); (**b**) basil cultivated with standard growth media substrate “Einheitserde” (E, Trial I) and 15% HFS-substrate (HFS, Trial II) irrigated with process water from the extensive aquaculture unit (EAU); and (**c**) comparison of basil grown in standard growth media substrate “Einheitserde” (E, Trial I) and 15% HFS (HFS, Trial II) irrigated with process water from the intensive aquaculture unit (IAU); different letters showing different groups (p < 0.05).

Growth of *O. basilicum* was different between pots filled with the commercial substrates (S1, S2), the standard growth media substrate (100% “Einheitserde”, E), and pots filled with 15% of the soil conditioner Humicacid Fiber-Substrate (HFS, Fig. 1a–c). The highest growth of basil was found in plants cultured in the control groups irrigated with commercial fertiliser and the original commercial pot substrates (S1, S2; Fig. 1a), followed by plants irrigated with intensive aquaculture effluents (HFS, Fig. 1c), and the lowest basil growth was found in plants watered with extensive fish process water (Fig. 3b). The growth performance of *O. basilicum* grown in pots with 15% Humicacid Fiber-Substrate and irrigated with intensive fish water was more comparable to the standard growth media substrate with 10 similar parameters out of 13 (Fig. 1c): plant height, green weight, dry weight, shoot length, shoot green weight, shoot dry weight, root length, root green weight, leaf number, and leaf length. Leaf width and leaf green weight were higher in plants cultured with HFS (Trial II), and only root dry weight was significantly higher in basil grown in the standard substrate (Trial I). Basil and HFS-Substrate (Trial II) irrigated with extensive aquaculture process water was only comparable in three parameters (root length, root green weight, root dry weight; Fig. 1b), whereas the standard substrate (E, Trial I) resulted in higher growth with other parameters.

### Nutrient compositions of the pot substrates

**Figure 2:**
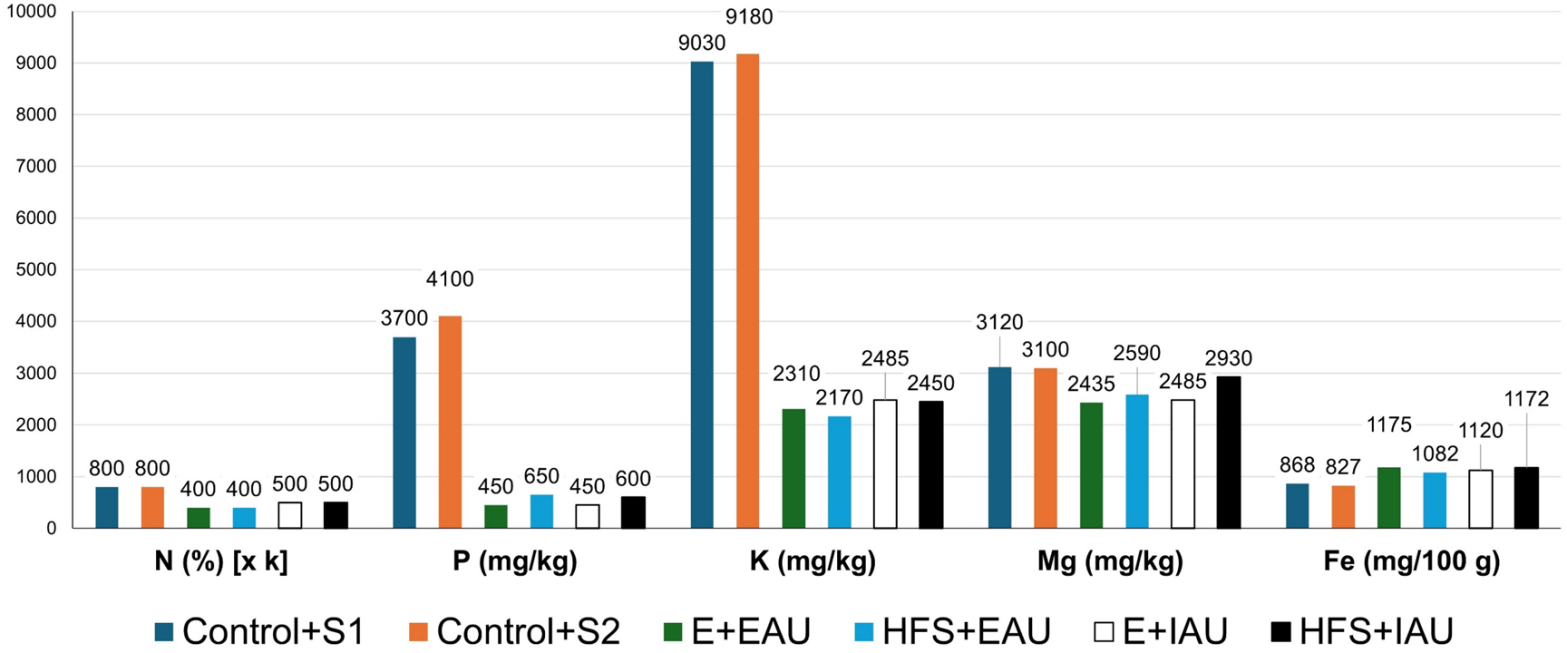
Comparison of gross nutrient composition (GNC) between control groups (S1-Trial I, S2-Trial II) with 100% Einheitserde substrate (+ commercial dried starter fertiliser) irrigated with commercial liquid fertiliser (Control + S1, S2), and pots filled with 100% Einheitserde (E, Trial I), and pots filled with 15% Humicacid Fiber-Substrate (HFS, Trial II) both irrigated with extensive (EAU) and intensive (IAU) aquaculture process water effluents; values of N (%) were multiplied (1 k).

Nutrient amounts of plant available nutrients (PAN, Table 3) in the control group substrates (S1, S2) were, in general, higher for NO_3_–N, P, K, B, Zn and Fe–EDTA compared to the EAU and IAU in the groups with the standard growth media substrate (E, Trial I) and Humicacid Fiber-Substrate (HFS, Trial II). In pots irrigated with intensive aquaculture effluents, the NO_3_–N levels were substantially higher by 39.9-fold in the standard substrate (E + IAU, Trial I), and by 38.4-fold in the HFS-substrate (HFS + IAU, Trial II) compared to the substrates irrigated with extensive fish process (EAU) water of the same trial. In PAN with intensive fish water and between trials (E + IAU, HFS + IAU), levels were comparable of P, K, B and Mo, and higher in the Einheitserde substrate (Trial I) of organic substance (7.7%), and NO_3_-N (23%), whereas higher values were found in HFS + IAU substrate (Trial II) of Zn (46%), NH_4_-N (41.7%), and Fe-EDTA (28.7%). Gross nutrient composition (GNC, Fig. 2) in HFS + IAU substrate was higher than E + IAU substrate in P with 25%, in Mg of 15%, and in Fe of 4.4%, whereas K was 1.4% higher in Einheitserde substrate with intensive process water (E + IAU), and N was comparable between trials.

**Table 3:** Comparison of garden pot plant available nutrients (PAN) between control groups (S1-Trial I, S2-Trial II) with 100% Einheitserde substrate (+ commercial dried starter fertiliser) irrigated with commercial liquid fertiliser (Control + S1, S2), and pots filled with 100% Einheitserde (E, Trial I), and pots filled with 15% Humicacid Fiber-Substrate (HFS, Trial II), both irrigated with extensive (EAU) and intensive (IAU) aquaculture process water effluents.

Parameters	Control + S1	Extensive (E + EAU)	Intensive (E + IAU)
Trial I: PAN standard growth media substrate (“Einheitserde”)			
Dry matter (%)	27.5	34.1	31.6
pH	5.9	7.1	7.1
P (mg/100 g)	61.3	31.2	15.0
K (mg/100 g)	457.0	47.0	11.0
Ca (mg/100 g)	1,118.8	976.0	1,235.2
B (mg/kg)	2.4	0.6	0.5
Zn (mg/kg)	9.6	3.0	2.9
Mo (mg/kg)	0.5	0.2	0.1
Organic substance (%)	46.0	23.2	36.6
NH_4_–N (mg/100 g)	1.0	0.6	0.7
NO_3_–N (mg/100 g)	40.3	1.0	39.9
Fe–EDTA (mg/kg)	1448.9	464.9	575.9

Trial II: PAN Humicacid Fiber-Substrate			
	Control + S2	Extensive (HFS + EAU)	Intensive (HFS + IAU)
Dry matter (%)	26.2	30.5	32.2
pH	5.7	7.0	7.2
P (mg/100 g)	71.4	23.3	15.0
K (mg/100 g)	452.0	12.0	11.0
Ca (mg/100 g)	1,178.3	858.6	1,149.3
B (mg/kg)	2.5	0.5	0.5
Zn (mg/kg)	10.7	6.2	5.4
Mo (mg/kg)	0.4	0.1	0.1
Organic substance (%)	31.3	26.6	33.8
NH_4_–N (mg/100 g)	0.7	0.8	1.2
NO_3_–N (mg/100 g)	49.5	0.8	30.7
Fe–EDTA (mg/kg)	1,623.9	977.9	807.9

### SPAD values of basil leaves

**Figure 3:**
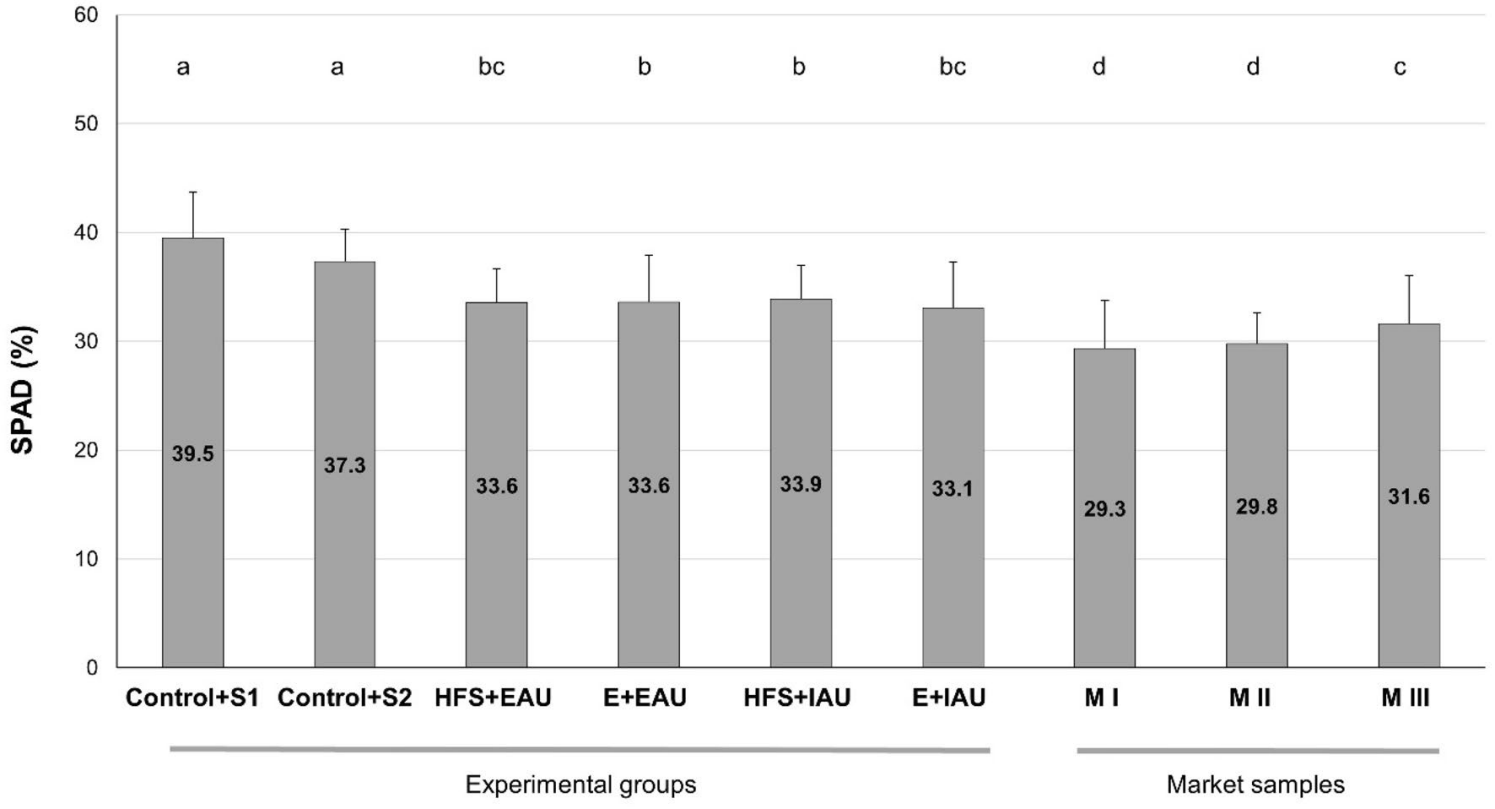
Comparison of *O. basilicum* leaf SPAD values (%) of experimental substrate and irrigation groups; with S1 (Trial I, n = 18) and S2 (Trial II, n = 18) denoting control groups (Control) with 100% Einheitserde substrate (+ commercial dried starter fertiliser) watered with commercial fertiliser, “E” denotes groups with 100% “Einheitserde” (Trial I, n = 18), and “HFS” denotes Humicacid Fiber-Substrate groups (Trial II, n = 18); EAU: plants irrigated with extensive aquaculture process water effluents (n = 18); IAU: plants irrigated with intensive aquaculture effluents (n = 18); M: market samples of basil leaves from local food chains: M I: sample from ALDI-Nord (Germany; n = 66); M II: sample from Netto Marken-Discount Stiftung & Co. KG (Germany; n = 66) and M III: sample from Netto ApS & Co. KG, Salling Group A/S (Germany, Denmark; n = 66); different letters showing different groups (p < 0.05), analysed by Kruskal–Wallis-ANOVA.

The relative chlorophyll content of *O. basilicum* leaves, measured as SPAD readings (%), showed comparable and significantly higher values in the control group plants (S1, S2) irrigated with commercial fertiliser (+ commercial dried starter fertiliser) and cultured in 100% standard growth media “Einheitserde” (Fig. 3) compared to plants grown in both substrates with aquaculture irrigations (EAU, IAU) and substrates (Humicacid Fiber-Substrate: HFS; Einheitserde: E). The SPAD levels were not significantly different between aquaponic groups (EAU, IAU); however, the samples of market plants (M) showed lower chlorophyll contents in M I (ALDI-Nord) and M II (Netto Marken-Discount Stiftung & Co. KG), whereas a sample from M III (Netto ApS & Co. KG, Salling Group A/S) was not significantly different in SPAD compared to HFS + EAU group and E + IAU group.

## Discussion

### Growth performance of *O. basilicum*

Basil growth was generally moderate (Tables 1, 2). *O. basilicum* can reach substantial heights of 75–95 cm under natural conditions and a longer growth period^26^; however, comparable heights in aquaponics were reported from deep-water culture (DWC) of 39.9 cm with production of Nile tilapia (*Oreochromis niloticus*)^27^ and in a decoupled system ranging from 46.78 to 55.75 cm under intensive production of *C. gariepinus* after 36 days^5^. Substantially better basil heights of 94.8–101.8 cm were found in decoupled aquaponics with 6–8 true leaves and a 4-cm shoot axis height at the transplanting stage^4,5^, in contrast to the earlier transplantation with lower heights (1.67 cm) and one pair of fully expanded leaves in the present study. In general, the number of leaves was reduced in all groups (Tables 1,2) as significantly higher leaf numbers of 493.7–518.0 were reported in decoupled aquaponics after 41 days^4^. Consequently, the intensive process water resulted in similar basil growth as observed from other aquaponic systems. This contrasts the low leaf number and reduced leaf dimensions in the EAU combined with HFS substrate, indicating low nutrient contents^28^ compared to the IAU (Table 2). Thus, the extensive aquaculture process water effluents with a portion of 15% Humicacid Fiber-Substrate was not able to replace the standard growth media and resulted in reduced plant growth due to nutrient deficiencies. Future studies should extend the transplanting time to a minimum of three weeks^4,5^ with four to five leaves^29^ to achieve optimal growth.

### Influence of the Humicacid Fiber-Substrate (HFS) on pot nutrient composition

The substitution of 15% standard media substrate (“Einheitserde”) by HFS in garden pots of the IAU increased the amount of plant available nutrients of Zn to 46%, of NH_4_–N to 41.7%, and of Fe–EDTA to 28.7%, and in gross nutrients of P to 25% and Mg to 15%, and in Fe of 4.4%, compared to the 100% standard growth media (Table 3, Fig. 2).

The amount of zinc was 1.9-fold higher in the IAU with HFS compared to the standard media substrate (Table 3). Zinc is known to improve chlorophyll formation in plants and could decrease interveinal leaf chlorosis and leaf deformations^30^ as was observed in the IAU plants with HFS by visual observation. The level of 5.4 mg/kg Zn in the HFS trial (IAU) was adequate; however, it should be increased to 10 mg/kg, as it was reported that Zn improved basil biomass production, nutrient uptake of K^+^ and Cu, and the chlorophyll index (SPAD)^31^. Both, the fish feed and the Humicacid Fiber-Substrate were sources of zinc, and the increase in stocking density and the proportion of the soil improver could increase the amount of zinc for the production of high-quality plants, knowing that up to 48% of it could be bound to the sludge^32^.

Plant available NH_4_–N was increased in the IAU Humicacid Fiber-Substrate group 1.7-folds compared to the standard substrate (Table 3), and it originated from organic substances. The addition of humic acid increased cation adsorption and the preference of NH_4_^+^ in a humic-montmorillonite clay mineral complex^33^. NH_4_^+^ is actively involved in plant growth, as studies have shown that the content changed significantly with the growing season, notably in clayey soils^34^. NH_4_^+^ formed pools in clay mineral interlayers, which are resistant to nitrification and can be available for plant growth through gradual release^35,36^. The combination of HFS with a higher proportion of humic acid and the higher proportion of NH_4_–N in the process water from the intensive fish production may have resulted in a fixation of NH_4_^+^ in the clay fraction of the substrate, which was obviously recovered for plant growth.

Plant available Fe–EDTA was 1.4-fold higher in the IAU with Humicacid Fiber-Substrate than in the substrate with “Einheitserde” of IAU (and 2.1-fold greater in EAU with HFS; Table 3) and was described as the most used synthetic chelating agent in fertilisers or as a supplement, e.g., 13% EDTA Fe at 2 mg/L with a three-week interval^37,38^, which is stable at pH 4.0–6.3^39^, as was observed in the process waters of the present study (Table 5). The Fe–EDTA origin in this experiment was unclear; however, only the Humicacid Fiber-Substrate might be a source by binding Fe with humic complexes under acidic conditions, as only in these groups the Fe–EDTA content increased. Fe–EDTA can form complexes with free metal cations and is able to prevent plant uptake of metals such as Zn, Cu, and Mn^39^, which was not evident in the case of Zn due to higher amounts in the HFS groups (Table 3). Iron and Fe–EDTA are essential for photosynthesis and a limiting factor in aquaponics, and the increased Fe–EDTA content might have prevented the interveinal chlorosis in basil^30^ that was observed in the plants of the HFS groups without chlorosis. The relatively comparable Fe contents in EAU and IAU (GNC) might be due to water leaching effects in the IAU caused by higher sedimenter cleaning intervals as a result of the higher stocking density.

The amount of phosphorus in the intensive group with HFS was about 1.3-folds higher in gross nutrients than in the standard substrate (Fig. 2). Sources of phosphorus included both, the fish feed and addition of HFS, which increased P in the aquaponic groups. Since the amount of P in the EAU and IAU groups with HFS was almost the same, i.e., independent of the fish stocking density, it can be assumed that the addition of HFS alone increased the amount of P. This is in accordance with the three-weight class production of *C. gariepinus*, which showed approx. the same phosphorus level inside the process water when the feed input ratio of extensive, semi-intensive and intensive production (feed ratio: 1:2:4) was calculated as 1.0:0.4:0.6 (staggered III production phase)^40^. Thus, an increased amount of phosphorus from adding HFS increased the growth of basil, which in combination with the other nutrients, enhanced the growth performance in the IAU compared with the standard substrate.

The amount of magnesium was slightly higher (≈ 1.2-fold) in the IAU with Humicacid Fiber-Substrate compared with the standard media substrate (Fig. 2). Fish feed as the main magnesium source can be excluded, as the Mg nutrient concentration ratios in the process water of African catfish production does not increase proportionally with increasing stocking density (Tables 4,5); magnesium can be bound inside the sludge to a higher extend, up to 16%^32^, and depending on levels of water exchange. This suggests that HFS was an additional source of Mg, and the 15% substitution of this soil conditioner for standard media minimally increased its proportion in the substrate.

**Table 4:** Comparison of chemo-physical water parameters (mean ± SD) of the extensive (EAU) and intensive (IAU) aquaculture units with *C. gariepinus* production; different letters show different groups (p < 0.05).

Parameters	Extensive (EAU)	Intensive (IAU)	*p*-value
O_2_ (mg/L)	7.3 ± 0.2^a^	6.3 ± 0.7^b^	0.001
O_2_ (%)	91.7 ± 2.5^a^	79.0 ± 8.3^b^	0.001
T (°C)	27.2 ± 0.5^a^	26.8 ± 0.4^b^	0.001
pH	6.6 ± 0.8^a^	5.3 ± 1.0^b^	0.001
Conductivity (µS/cm)	1022.9 ± 91.2^b^	1603.6 ± 230.6^a^	0.001
Salinity (‰)	0.5 ± 0.0^b^	0.8 ± 0.1^a^	0.001
Redox potential (mV)	166.9 ± 19.8^b^	185.1 ± 30.2^a^	0.004
NH_4_-N (mg/L)	0.38 ± 0.278^b^	1.92 ± 1.531^a^	0.001
NO_2_-N (mg/L)	0.28 ± 0.15^b^	1.6 ± 1.6^a^	0.001
NO_3_-N (mg/L)	241.61 ± 109.49^b^	501.64 ± 70.79^a^	0.001
TON (mg/L)	241.88 ± 109.58^b^	503.28 ± 71.64^a^	0.001
PO_4_^3^-P (mg/L)	17.56 ± 4.60^a^	13.80 ± 9.91^a^	0.464
K^+^ (mg/L)	7.00 ± 2.50^b^	11.73 ± 3.39^a^	0.001
Mg^2+^ (mg/L)	17.49 ± 3.99^a^	18.93 ± 4.31^a^	0.437
Ca^2+^ (mg/L)	109.88 ± 27.11^b^	199.76 ± 23.11^a^	0.001
Fe^2+^ (mg/L)	0.02 ± 0.01^a^	0.02 ± 0.01^a^	0.238
SO_4_^2^-S (mg/L)	80.55 ± 16.06^b^	97.47 ± 18.33^a^	0.002

**Table 5:** Hydroponic physico-chemical water parameters (means ± SD) in the fertiliser control tank (Control) and the experimental aquaculture process water tanks (extensive: AET-E; intensive: AET-I) with light parameters between planting tables of Humicacid Fiber-Substrate (HFS) and standard growth media substrate (Einheitserde, E) trial (light intensity, PPFD); different letters showing different groups (p < 0.05; *p*-I: significance value (*p*) between Control & EAU; *p*-II: between Control & IAU; *p*-III: between EAU and IAU).

Parameters	Control	Extensive (AET-E)	Intensive (AET-I)	*p*-I	*p*-II	*p*-III
O_2_ (mg/L)	8.6 ± 0.2^a^	8.6 ± 0.2^a^	8.6 ± 0.3^a^	0.702	0.702	0.702
O_2_ (%)	103.9 ± 1.5^a^	104.0 ± 1.1^a^	103.5 ± 2.6^a^	0.887	0.887	0.887
T (°C)	24.6 ± 1.1^a^	25.1 ± 1.2^a^	25.0 ± 1.3^a^	0.278	0.479	0.924
pH	6.0 ± 0.1^a^	5.9 ± 0.1^b^	6.0 ± 0.2^a^	0.025	1.000	0.002
Conductivity (µS/cm)	2,011.8 ± 72.4^a^	995.7 ± 89.4^c^	1,708.2 ± 173.2^b^	0.001	0.001	0.001
Salinity (‰)	1.0 ± 0.0^a^	0.5 ± 0.0^c^	0.9 ± 0.1^b^	0.001	0.001	0.001
Redox potential (mV)	185.4 ± 15.6^a^	176.3 ± 9.3^a^	181.3 ± 17.4^a^	0.054	0.536	0.407
NH_4_–N (mg/L)	11.91 ± 9.19^a^	0.43 ± 0.24^b^	1.35 ± 0.96^ab^	0.001	0.078	0.057
NO_2_–N (mg/L)	1.09 ± 2.28^a^	0.05 ± 0.07^a^	0.22 ± 0.23^a^	0.095	0.095	0.095
NO_3_–N (mg/L)	116.47 ± 16.07^a^	57.69 ± 9.08^b^	144.00 ± 20.15^a^	0.015	0.125	0.001
TON (mg/L)	117.57 ± 14.99^a^	57.72 ± 9.07^b^	144.22 ± 20.05^a^	0.015	0.125	0.001
TDN (mg/L)	128.29 ± 16.51^a^	53.30 ± 18.74^b^	145.35 ± 19.76^a^	0.003	0.637	0.001
PO_4_^3^–P (mg/L)	64.56 ± 17.01^a^	17.25 ± 7.28^b^	24.42 ± 15.73^b^	0.001	0.003	0.805
K^+^ (mg/L)	320.41 ± 36.05^a^	7.98 ± 2.09^b^	18.23 ± 5.40^b^	0.001	0.020	0.063
Mg^2+^ (mg/L)	47.84 ± 5.57^a^	21.86 ± 4.72^b^	27.15 ± 8.39^b^	0.001	0.011	0.255
Ca^2+^ (mg/L)	96.16 ± 14.34^b^	129.90 ± 28.18^b^	232.34 ± 47.42^a^	0.064	0.001	0.017
Fe^2+^ (mg/L)	0.01 ± 0.01^b^	0.02 ± 0.01^a^	0.02 ± 0.01^a^	0.013	0.040	1.000
SO_4_^2^–S (mg/L)	68.14 ± 8.46^a^	30.34 ± 3.51^b^	37.91 ± 14.05^b^	0.001	0.005	0.633
Humicacid Fiber-Substrate (HFS)						
Light intensity (lx)	330.1 ± 265.5^a^	323.7 ± 245.0^a^	382.9 ± 263.6^a^	0.695	0.695	0.695
PPFD (µmol/m^2^s)	462.0 ± 344.0^a^	516.0 ± 390.1^a^	510.9 ± 384.4^a^	0.861	0.861	0.861
Standard growth media substrate (“Einheitserde”)						
Light intensity (lx)	372.4 ± 243.1^a^	354.7 ± 252.1^a^	329.7 ± 236.1^a^	0.893	0.893	0.893
PPFD (µmol/m^2^s)	585.0 ± 419.2^a^	582.5 ± 405.5^a^	551.1 ± 403.0^a^	0.951	0.951	0.951

### Chlorophyll content of basil leaves

SPAD readings (chlorophyll content) were significantly higher in control group plants (S1, S2) due to the use of the commercial fertiliser (Fig. 3). In contrast, SPAD values of basil from aquaponics with HFS and standard substrates were not significantly different (comparable) and higher compared with those taken from supermarkets, except sample MIII (Netto ApS & Co. KG; Fig. 3). SPAD levels in the aquaponics and the market samples were lower than reported for basil irrigated with organic fertiliser (chicken manure) of 35.18%, and the SPAD of the control group plants were closer to basil fertilised with spores of mycorrhizal fungi and bacteria of 40.27%^41^. Lower leaf chlorophyll content was found in basil cultivated in aquaponics with crayfish (*Procambarus* spp.), with 29.3 SPAD and hydroponics with 28.7 SPAD^25^, and in aquaponics with Nile tilapia (*O. niloticus*) of 23.2% and hydroponics of 31.7%^27^. *O. basilicum* cultivated under different daily light intervals showed a lower SPAD value of 25.7% (≈ 7 mol/m^2^ d) and a higher SPAD level of 34.1% (≈ 15 mol/m^2^ d) in a nutrient-film technique (NFT) hydroponic system^42^. Thus, basil cultivated under aquaponics cultivated in HFS-substrate with effluents from extensive and intensive *C. gariepinus* aquaculture had a higher leaf chlorophyll content than aquaponics with other aquatic animals and showed a better chlorophyll level than samples from the supermarkets, as an indication of high-quality *O. basilicum* production that was equivalent to the market samples in terms of leaf colour (SPAD).

## Conclusions

The substitution of the standard growth media substrate (“Einheitserde”) with 15% of the soil conditioner Humicacid Fiber-Substrate resulted in an improved growth of *O. basilicum* in pot cultivation, similar to 100% standard substrate in combination only with intensive effluents from *C. gariepinus* production. It also showed a better chlorophyll content (SPAD) than basil samples from local markets. Therefore, the application of Humicacid Fiber-Substrate in aquaponics with African catfish is a new option to reduce peat without compromising basil quality.

The combination of aquaculture process water from the intensive stocking of African catfish and 15% Humicacid Fiber-Substrate was effective and increased levels of several nutrients, including zinc, NH_4_–N, Fe–EDTA, phosphorus, and magnesium. However, to ensure higher plant quality and growth, zinc should be increased 10 mg/kg, and iron could be added in the form of chelate with 2.5 mg/L every three weeks. Further adaptations are possible by using resources from the agricultural circular economy for the nutrient composition of the soil conditioner Humicacid Fiber-Substrate, or by increasing the substitution rates of the peat-based standard substrate with Humicacid Fiber-Substrate above 15%. This might further improve basil growth and quality while reducing the amount of peat with its negative impacts through peat mining and increased CO_2_ emissions.

## Methods

All methods were carried out in accordance with relevant guidelines and regulations.

### Experimental facility

**Figure 4:**
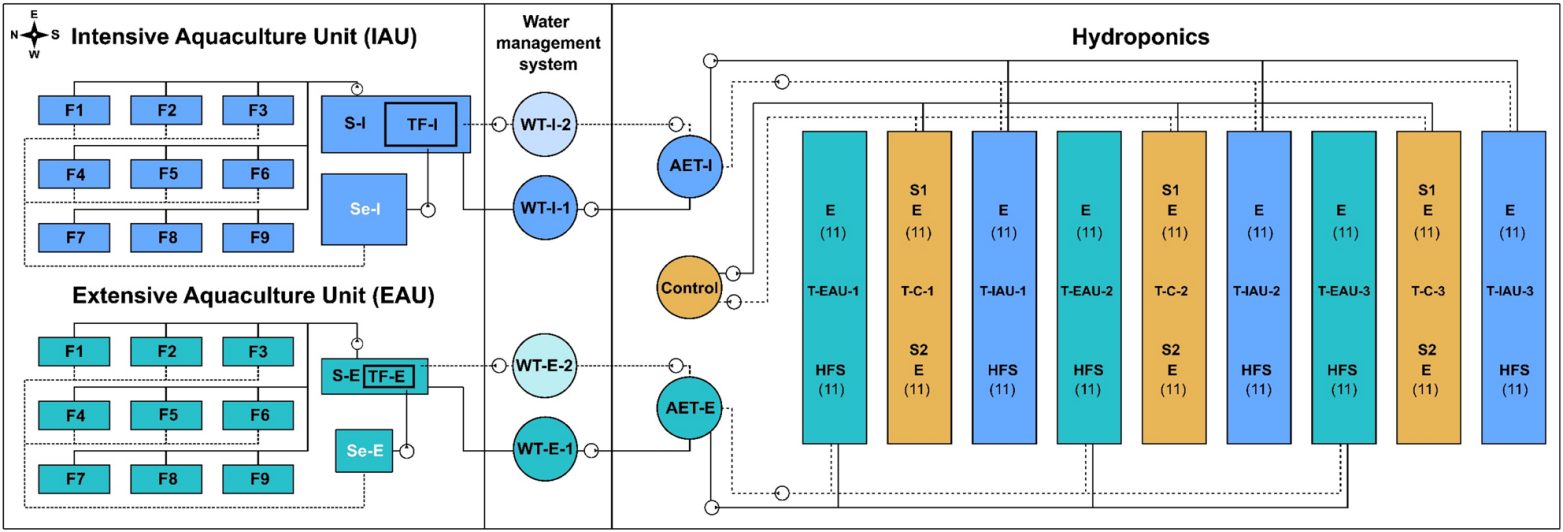
Schematic illustration of the experimental design in the FishGlassHouse with aquaculture section: intensive aquaculture unit (IAU) with nine fish tanks (F1-9), sedimenter (Se-I), sump (S-I) and trickling filter (TF-I); extensive aquaculture unit (EAU) with nine fish tanks (F1-9), sedimenter (Se-E), sump (S-E), and trickling filter (TF-E); water management system with tanks supplying waste water to hydroponics (WT-I-1 of IAU; WT-E-1 of EAU), and discharging waste water tanks (WT-I-2 of IAU; WT-E-2 of EAU); hydroponics unit with aquaculture effluent tanks for the IAU (AET-I) and EAU (AET-E), dissolved fertiliser liquid tank for control (Control) and nine ebb-and-flood planting tables corresponding to aquaculture effluent and control fertiliser tanks (T-EAU-1/3, T-C-1/3, T-IAU-1/3), substrate variants were symbolised from Trial I: with control as S1 and "Einheitserde" as E, and Trial II: control (S2) and “Humicacid Fiber-Substrate” as HFS with used pot numbers (11) for analytics in brackets. Fluid supply pipes are shown solid and return pipes are drawn dashed.

The experiment was carried out at the aquaponics experimental facility “The FishGlassHouse” in spring 2018 (April to June) for in total 49 days, using the extensive (EAU, 100 m^2^) and intensive recirculating aquaculture units (IAU, 100 m^2^; PAL Anlagenbau GmbH, Germany) and one hydroponics unit (100 m^2^) in a VENLO greenhouse (GTW Gewächshaustechnik Werder GmbH, Germany) at the University of Rostock (UoR), Faculty of Agricultural and Environmental Sciences, Northern Germany in Mecklenburg-Western Pomerania (GPS: latitude: 54.075714, longitude: 12.096591, Fig. 4).

The aquaculture units consisted of nine fish tanks (F 1–9, water volume 1 m^3^; Fig. 4) arranged in triplicates (3 × 3) for staggered fish production of three different fish weight classes, one solids separation unit / sedimenter (Se-E in EAU: 1.1 × 1.2 × 0.9 m and 1.2 m^3^; Se-I in IAU: 1.5 × 1.3 × 0.9 m and 1.7 m^3^), nitrifying trickling filters (TF-E in EAU: 2.9 m^3^; TF-I in IAU: 11.8 m^3^), and communicating sumps (S-E in EAU: 1.6 m^3^; S-I in IAU: 4.0 m^3^)^7^. Aquaculture process water was transferred semi-continuously via a water management system and tanks (WT-E-1/2 in EAU and WT-I-1/2 in IAU approx. 1,500 L) into the hydroponic cabin with corresponding aquaculture tanks for the EAU (AET-E), IAU (AET-I), and an additional hydroponics control group tank (Control) with a fertiliser solution (each approx. 1,000 L). For semi-coupled aquaponics conditions, nutrient solutions were pumped back from the hydroponics unit via water management system to the extensive and intensive aquaculture units twice a week.

### Fish production

All fish were delivered in January 2018 (30 g/fish initial weight) from a local fish farm (Fischzucht Abtshagen GmbH & Co. KG, Germany) and stocked in the extensive (33.8 fish/m^3^ and tank) and intensive (132.4 fish/m^3^ and tank) aquaculture units (EAU, IAU). Three weight classes of *C. gariepinus* were held staggered in triplicates with mean initial weights of 629.2 ± 473.2 g for the EAU and 655.0 ± 438.9 g for the IAU (p = 0.653), and final weights of 806.7 ± 457.2 g for EAU and 838.5 ± 477.1 g for IAU (p = 0.966) from 25.04.2018 to 12.06.2018 (49 days). The initial and final stocking density for EAU was: 18.1 ± 13.6 kg/m^3^, and 24.9 ± 15.8 kg/m^3^, and for IAU: 73.4 ± 56.8 kg/m^3^ (p = 0.014), and 89.5 ± 55.6 kg/m^3^ (p = 0.004), with initial and final biomasses in EAU of 163.0 kg and 223.9 kg (mortality: 2.63%); and for IAU of 660.2 kg and 805.8 kg (mortality; 1.34%; initial fish biomass ratio: 4.1). Fish were fed by automatic feeders according to a standard commercial feeding protocol with a feed load of 80% as recommended (PAL GmbH, Germany). The feed used was Alltech Coppens Special Pro EF 4.5 mm (The Netherlands) with levels of 42.0% protein, 13% fat, 1.5% crude fibre, 7.6% ash, 1.02% phosphorous, 1.9% calcium, 0.3% sodium, 60 mg/kg iron, 5 mg/kg iodine, 5 mg/kg copper, 20 mg/kg magnesium, and 60 mg/kg zinc. Length was measured with a hand ruler and weights by scales (SBS-PF-A150/20, Steinberg Systems, Poland; PCE-BS 300, PCE Deutschland GmbH, Germany). Accumulated fish performance was for the EAU: FCR 0.92 ± 0.14 and specific growth rate (SGR) 0.92 ± 0.51%/d, and for the IAU: FCR 1.25 ± 0.65 and SGR: 0.83 ± 0.55%/d (FCR: p = 0.167; SGR: p = 0.423; p > 0.05) with a mean feed use of 6.5 ± 2.9 kg for the EAU and 22.8 ± 11.0 kg for the IAU (p = 0.001) by staggered production, and a total feed use of 58.2 kg for EAU, and 182.1 kg for IAU, this corresponds to a daily feed quantity for EAU of 1.2 kg feed/day, and for IAU of 3.7 kg feed/day.

### Plant production

*O. basilicum* seeds (variation “Genovese”, Kiepenkerl, Germany; N = 350) were germinated in 100% standard media substrate (“Einheitserde, Nullerde Typ 0”) in the FishGlassHouse and transferred randomly to the hydroponics (initial height: 1.67 ± 0.2 cm; Fig. 4) with an experimental duration of 45 days (24.04.2018 to 07.06.2018). The plants were distributed on nine ebb-and-flood tables (3.05 × 1.01 m, Otte Metallbau GmbH & Co. KG, Germany), divided into three blocks, with originally 15 plant pots per table and trial (270 plants in total), of these where 11 pots per substrate and irrigation group randomly selected for data analysis, 33 pots each for S1, S2, E-EAU, E-IAU, HFS-EAU, HFS-IAU (total 198 pots); with a spacing of 10 cm (Fig. 4).

Control group plants (S1-Trial I, S2-Trial II) grew in pots with 100% conventional standard growth media substrate (one pot with 250 g, white peat 80%: 0–10 mm and clay 20%, pH 5.5–6.5; Einheitserde EE-Typ 0 “Nullerde” after Fruhstorfer^12^; Einheitserdewerke Patzer, Patzer Erden GmbH, Sinntal‒Altengronau, Germany) with addition of commercial starter fertiliser (Grönfingers GmbH, Germany) with P: 750 mg/kg, K: 2020 mg/kg, Mg: 1815 mg/kg, N: 0.72%, Fe 718.81 mg/100 g of dry matter^43^ and irrigated by commercial hydroponic fertiliser solution.

Plants grown under aquaponic conditions without fertiliser (EAU; IAU) were potted in a substrate mixture of ≈ 15% soil conditioner Humicacid Fiber-Substrate (HFS-Trial II)^18^ with a mean weight of 37.55 g = 15.05% (n = 33) and ≈ 85% standard growth media substrate (211.89 g = 84.94%, n = 11; Einheitserde EE‒Type “0 Nullerde” after Fruhstorfer^12^) with P: 500 mg/kg, K: 3120 mg/kg, Mg: 3125 mg/kg, N: 0.42% and Fe 1217.80 mg/100 g of dry matter^43^. Substrate of plants grown in standard growth media were cultivated in 100% “Einheitserde” (E, Trial I), without commercial starter fertiliser, and had gross nutrients of P: 350 mg/kg, K: 2615 mg/kg, Mg: 2565 mg/kg, N: 0.35% and Fe: 1131.80 mg/100 g of dry matter^43^.

The Biologically pure three-phase natural-fertiliser Humicacid Fiber-Substrate (HFS, old name “BIOHUMIN^®^”) consisted of a mineral-humus substrate mix made from natural substances without pollutants and without synthetic chemicals. The media contained the following three main ingredients: 5–30% lime powder (mussel lime), 50–70% magma stone powder, and 50–30% milled tertiary raw lignite, with different minerals (e.g. calcite, illite, montmorillonite) and metal oxides. The fibre matter further consisted of insoluble sulphates, trace elements, humic substances, various N-compounds, lignin, cellulose, plant resins, and plant waxes with 1/3 nutrient humus content and 2/3 permanent humus content. The substrate mixture used in this experiment originates from patent WO1993010061A1 of 1992^18^.

In the hydroponics, each nutrient solution tank (Control, EAU: AET-E, IAU:AET-I; Fig. 4) with its associated recirculating system (Fackler Gewächshaustechnik, Germany) was equipped with an automated pH-controller (Bluelab Corporation Limited, New Zealand) for pH adjustment at 6.0 (± 0.2); for increasing pH, “pH + up” solution (28.2% potassium hydroxide) was used, and for decreasing pH, “pH-bloom” solution was used (59% phosphoric acid; Advanced Hydroponics of Holland B.V., The Netherlands). The nutrient tank of the control group was filled with commercial fertiliser Universol^®^ Orange 16-5-25 + 3.4 MgO + TE with 16% N (N–NH_4_: 5.2%; N–NO_3_: 10.4%), 5.0% P_2_O_5_, 25% K_2_O, 3.4% MgO, 0.10% Fe (EDTA-chelate), 0.04% Mn (EDTA-chelate), 0.01% B, 0.010% Cu (EDTA-chelate), 0.001% Mo, 0.010% Zn (EDTA-chelate; ICL Specialty Fertilizers, ICL SF Germany & Austria) adjusted to an electrical conductivity of approx. 2,000 ± 50 μS/cm. Tanks were ventilated by an air membrane pump (Mistral 4000, Aqua Medic GmbH, Germany). Two times per week (Monday, Thursday), the aquaculture process water effluent of aquaponics experimental groups was exchanged with new aquaculture effluents from the fish production units (EAU, IAU) via a water management system and pumped back to aquaculture units for semi-coupled aquaponics conditions, and the evaporated nutrient water of the control group tank (Control; Fig. 4) was replaced with freshwater as required and mixed with fertiliser. Aquaculture effluent water and commercial nutrient solution was pumped four times per day via an automated clock (Fackler Gewächshaustechnik, Germany) on ebb-and-flood tables with flooding up to a height of about 5 cm water level (4 min flood and 4 min ebb interval).

Plant growth was measured in length (hand ruler) and weight (Analytical balance ATX224, Shimadzu Corporation, Japan; PCE-BS 300, PCE Deutschland GmbH, Germany), and dry weight was determined in a drying oven (Memmert UN750, Germany) after drying for 23 h at 60 °C and one hour at 100 °C. For SPAD levels (each n = 66), leaves were taken from local superstores: samples M I: ALDI-Nord (ALDI GmbH & Co. Kommanditgesellschaft, Germany); M II: Netto Marken-Discount Stiftung & Co. KG (Germany) and M III: sample from Netto ApS & Co. KG, Salling Group A/S (Germany, Denmark).

### Physical and chemical parameters

Physical water parameters were taken daily (threefold), from the sumps during the week in the aquacultural units (EAU, IAU) and hydroponic tanks, including temperature (°C), dissolved oxygen (DO, mg/L), oxygen saturation (%), conductivity (EC, µS/cm), pH, and redox potential (mV) with a HQ40D multimeter (Hach Lange GmbH, Germany). Photosynthetic photon flux density (PPFD, µmol/m^2^s) was measured by a Lightscout-3415FSE Solar Electric Quantum Meter (Spectrum Technologies, Inc., Aurora, IL, USA), and light intensity by digital illuminance/light meter LX1330B (Dr. Meter). SPAD was recorded from six plants per group (n = 18) by a Chlorophyll Meter SPAD-502-Plus (Konica Minolta, Inc., Marunouchi, Japan).

Chemical water parameters were taken twice per week (Monday, Thursday; three-fold) and analysed by a Gallery™ Automated Photometric Analyzer (Thermo Fisher Scientific, Waltham, MA, USA) for ammonium (NH_4_^+^), nitrite (NO_2_^−^), nitrate (NO_3_^−^), phosphate (PO_4_^3−^), potassium (K^+^), magnesium (Mg^2+^), calcium (Ca^2+^), iron (Fe^2+^), and sulphate (SO_4_^2−^). TON (total oxidized nitrogen), as N and nitrate, was measured by calculation (TON-nitrite) and by the colorimetric hydrazine method (D08896_01^©^ 2020 Thermo Fisher Scientific Inc.). At the end of the experiment, pooled samples of three pots were taken and examined for gross and plant available nutrient analysis by LMS-LUFA^43^ (Landwirtschaftliche Untersuchungs- und Forschungsanstalt der LMS Agrarberatung GmbH, Rostock, Germany).

### Chemo-physical water parameters of aquaculture units

Water parameters of aquaculture units (EAU, IAU) were comparable in PO_4_^3^-P, Mg^2+^, and Fe^2+^. Every nitrogen compound such as nitrate were significantly higher with intensive stocking of *C. gariepinus*, while K^+^ was slightly higher, and Ca^2+^ was almost twice as high than extensive stocking (Table 4).

### Chemo-physical water parameters of hydroponic irrigation tanks

Fertilised water in the control tank showed the significantly highest levels of conductivity (2,011.8 ± 72.4 µS/cm), followed by the IAU (1,708.2 ± 173.2 µS/cm) and the EAU (995.7 ± 89.4 µS/cm; Table 5). Levels of NO_3_–N were comparable between the control (116.47 ± 16.07 mg/L) and the intensive process water (144.00 ± 20.15 mg/L), followed by the water of the extensive aquaculture unit (57.69 ± 9.08 mg/L). In the control, higher levels of PO_4_^3^–P were found (64.56 ± 17.01 mg/L) and were similar in both aquaponic groups (IAU: 24.42 ± 15.73 mg/L; EAU: 17.25 ± 7.28 mg/L), as well as Mg^2+^ having the highest value in the control (47.84 ± 5.57 mg/L) and similar levels in the IAU (27.15 ± 8.39 mg/L) and EAU (21.86 ± 4.72 mg/L). Global light parameters in hydroponics between the standard growth media substrate Einheitserde (E, Trial I) and Humicacid Fiber-Substrate (HFS, Trial II) were not significantly different. Light intensity in HFS was 345.6 ± 255.4 lx, and in the standard growth media 352.3 ± 240.6 lx (p = 0.570, n = 63). Photosynthetic active light (PPFD) in HFS was 496.3 ± 368.2 µmol/m^2^s, and in the standard growth media 572.9 ± 402.9 µmol/m^2^s (p = 0.217, n = 63).

### Statistical analysis

Statistical analyses were carried out by SPSS version 29^44^ and Excel^®^^45^ (p ≤ 0.05; two-tailed). Analyses were performed by one-way ANOVA at normal distribution (Shapiro–Wilk test) and post hoc by variance homogeneity with the Tukey-HSD test, or, at variance inhomogeneity with the Dunnett-T3 test. Non-normally distributed data and different sample sizes were tested by a nonparametric Kruskal–Wallis-ANOVA and Bonferroni correction. Two groups were analysed by *t*-test (normally distributed); otherwise, the Mann–Whitney-U test was applied. The sample sizes were determined *a-priori* using the G*Power programme^46^ and the experimental design was built in the FishGlassHouse accordingly. Specific growth rate of fish (SGR) was calculated as: SGR% = [((lnW*f* − lnW*i*)/t) × 100], W*f* was wet weight determined at t1; W*i*, wet weight determined at t0, and t the number of days; feed conversion ratio (FCR): fish feed quantity (g)/weight gain (g).

### Ethics approval

The study was approved by Landesamt für Landwirtschaft, Lebensmittelsicherheit und Fischerei Mecklenburg-Vorpommern—Veterinärdienste und Landwirtschaft.

## Data Availability

All data obtained or analysed in this study are included in the publication. Additionally, the analyses can be requested from the corresponding author. No datasets were generated or analysed during the current study.

## Abbreviations

C: Control group, irrigated with commercial fertiliser solution
DWC: Deep-Water Culture (hydroponic cultivation technology)
E: Standard growth media substrate "Einheitserde"
EAU: Extensive Aquaculture Unit
EC: Electrical Conductivity (µS/cm)
FCR: Feed Conversion Ratio (fish feed [g]/weight gain [g])
GNC: Gross Nutrient Composition
HFS: Substrate mix, with 15% Humicacid Fiber-Substrate and 85% E-Substrate
IAU: Intensive Aquaculture Unit
M I: Sample from ALDI-Nord (Germany, food chain company)
M II: Sample from Netto Marken-Discount (Germany, food chain company)
M III: Sample from Netto ApS & Co. KG, Salling Group A/S (Germany, Denmark, food chain company)
n: Sample size
NFT: Nutrient-Film Technique (hydroponic cultivation technology)
PAN: Plant Available Nutrients
PPFD: Photosynthetic Photon Flux Density (µmol/m^2^s)
S1: Commercial control substrate with 100% E-Substrate + starter fertiliser
S2: Commercial control substrate with 100% E-Substrate + starter fertiliser
SGR: Specific Growth Rate (% fish growth/d)
SPAD: "Soil Plant Analysis Development", chlorophyll measuring unit (%)
Trial I: Basil cultivation in E-substrate, irrigated by EAU and IAU, compared to C + S1
Trial II: Basil cultivation in HFS, irrigated by EAU and IAU, compared to C + S2
